# Assessment of Myoelectric Controller Performance and Kinematic Behavior of a Novel Soft Synergy-Inspired Robotic Hand for Prosthetic Applications

**DOI:** 10.3389/fnbot.2016.00011

**Published:** 2016-10-17

**Authors:** Simone Fani, Matteo Bianchi, Sonal Jain, José Simões Pimenta Neto, Scott Boege, Giorgio Grioli, Antonio Bicchi, Marco Santello

**Affiliations:** ^1^Centro di Ricerca E. Piaggio, Università di Pisa, Pisa, Italy; ^2^Neural Control of Movement Laboratory, School of Biological and Health Systems Engineering, Arizona State University, Tempe, AZ, USA; ^3^PES Institute of Technology, Bangalore, India; ^4^Pontifical Catholic University of Minas Gerais, Belo Horizonte, Brazil; ^5^Advanced Robotics Department, Istituto Italiano di Tecnologia, Genova, Italy

**Keywords:** prosthetics, assistive robotics, rehabilitative robotics, myoelectric control, kinematics

## Abstract

Myoelectric artificial limbs can significantly advance the state of the art in prosthetics, since they can be used to control mechatronic devices through muscular activity in a way that mimics how the subjects used to activate their muscles before limb loss. However, surveys indicate that dissatisfaction with the functionality of terminal devices underlies the widespread abandonment of prostheses. We believe that one key factor to improve acceptability of prosthetic devices is to attain human likeness of prosthesis movements, a goal which is being pursued by research on social and human–robot interactions. Therefore, to reduce early abandonment of terminal devices, we propose that controllers should be designed so as to ensure effective task accomplishment in a natural fashion. In this work, we have analyzed and compared the performance of three types of myoelectric controller algorithms based on surface electromyography to control an underactuated and multi-degrees of freedom prosthetic hand, the SoftHand Pro. The goal of the present study was to identify the myoelectric algorithm that best mimics the native hand movements. As a preliminary step, we first quantified the repeatability of the SoftHand Pro finger movements and identified the electromyographic recording sites for able-bodied individuals with the highest signal-to-noise ratio from two pairs of muscles, i.e., flexor digitorum superficialis/extensor digitorum communis, and flexor carpi radialis/extensor carpi ulnaris. Able-bodied volunteers were then asked to execute reach-to-grasp movements, while electromyography signals were recorded from flexor digitorum superficialis/extensor digitorum communis as this was identified as the muscle pair characterized by high signal-to-noise ratio and intuitive control. Subsequently, we tested three myoelectric controllers that mapped electromyography signals to position of the SoftHand Pro. We found that a differential electromyography-to-position mapping ensured the highest coherence with hand movements. Our results represent a first step toward a more effective and intuitive control of myoelectric hand prostheses.

## 1 Introduction

The introduction of myoelectric devices has profoundly modified prosthetics, especially in upper-limb amputation. Upper-limb myoelectric devices use electrical activity associated with muscle contraction (electromyographic signals, EMG) recorded from residual muscles in individuals with upper-limb loss to control movements of terminal devices, i.e., arm and/or hand prostheses. At the same time, electric actuators address some of the drawbacks of body-powered devices, such as heavy harness and limited functionality (Van Lunteren et al., 1983). Furthermore, myoelectric prostheses can offer better esthetics, greater pinch strength, and ease of operation (Biddiss and Chau, 2007). However, despite these advantages relative to body-powered devices, the rejection rate of myoelectric prostheses remains very high. Specifically, more than 23% of myoelectric prosthesis users abandon their devices (Biddiss and Chau, 2007). The main causes for abandonment of myoelectric devices include higher maintenance needs, greater costs, heavier weight, and low intuitiveness of control. These limitations suggest that improving intuitiveness of myoelectric control might contribute to reducing the rejection rate of myoelectric devices. In the present study, we propose that this objective could be attained by improving the correspondence between natural and prosthetic hand movements. The underlying assumption of our proposition is that individuals with upper-limb loss might prefer using a prosthetic device that can be controlled using similar muscle activation patterns underlying the control of the native hand, as opposed to being forced to learn abstract EMG patterns to fit the design of the terminal device. This approach could thus play a crucial role for increasing both acceptability and the sense of embodiment, i.e., the sense of the prosthesis becoming part of the user’s body by transitioning from an extracorporeal to a corporeal structure (Fraser, 1984; Scarry, 1994; Murray, 2008).

Within the above conceptual framework, anthropomorphism may be an additional factor to be considered to improve users’ acceptance of myoelectric prostheses, a concept that has been extensively studied in the field of human–robot interaction (Bartneck et al., 2009; Riek et al., 2009; Dragan and Srinivasa, 2014). Specifically, myoelectric prostheses represent a category of bidirectional human–robot interactions, as users control the terminal device through muscle activation. To improve effectiveness of prostheses performance, an additional aspect to consider is the correct identification of body sites for the acquisition of EMG through surface electrodes (Micera et al., 2010). This is an important factor to ensure optimal EMG signal quality, which in turn is necessary to improve control of the terminal device, while trying to compensate for unavoidable issues such as sweat or movement of the electrode relative to the target site.

In the literature on myoelectric prostheses, one can distinguish two approaches: (1) minimalistic mapping for standard one degree of freedom (DOF) hand prostheses, where it is customary to use two EMG electrodes located on an antagonistic muscle pair, e.g., residual wrist flexor and extensor muscles, to control the closing and opening of the prosthesis (Ajoudani et al., 2013) and (2) mapping EMG signals from multiple muscles to multi-DOFs devices [for a survey on the usage of multi-sEMG signals for robotic hand control, the reader is referred to Ison and Artemiadis (2014)]. Regarding (1), Ajoudani et al. (2012) examined the incorporation of the users’ intent in the control command not only in terms of desired motion or equilibrium position but also as stiffness profile estimated on the master side through a suitable human–machine interface. This approach, called Tele-impedance, was proven to be a viable solution also to overcome stability problems in force reflecting teleoperation and enable a more human-like task execution (Ajoudani et al., 2012). Ajoudani et al. (2014) used the major finger antagonist muscle pair, i.e., the m. extensor digitorum communis (EDC) and m. flexor digitorum superficialis (FDS), to map the reference commands by leveraging upon a modified hyperbolic tangent shape. With regard to (2), interfacing EMG signals from multiple residual muscles with a multi-DOF systems requires a direct mapping from multi-EMG patterns to control commands, and this is often accomplished by using machine learning (ML) techniques. However, one of the main issues for a deployment of these methods in clinical settings is its low reliability (Biddiss and Chau, 2007). To mitigate this drawback, the concept of simultaneous and proportional (s/p) myocontrol was proposed by Jiang et al. (2009), which enables mapping of EMG signals to activation of several DOFs based on regression techniques, rather than a classifier (Ison and Artemiadis, 2015). Another valuable contribution in this area is the idea of interactive and incremental learning: since calibration of ML methods usually employs a one-shot initial phase to strengthen its robustness, the function approximation can be further refined after this phase, thus leading to a model update that can correct control instabilities (González and Castellini, 2013); for further details on these topics, the reader is referred to Santello et al. (2016). A different approach consists of invasive myo-controlled prosthetic devices, which combines surgery, bionic reconstruction, and engineering, in some cases, enhanced by sensory feedback [see, for example, Ortiz-Catalan et al. (2014), Raspopovic et al. (2014), and Aszmann et al. (2015)].

In the present work, we focused on the control of the SofHand-Pro (SH-P), the prosthetic myoelectric version of an existing robotic hand, the Pisa/IIT SoftHand (SH) (Catalano et al., 2012, 2014; Ajoudani et al., 2014). The SH is a humanoid robotic hand that combines the concept of kinematic motor synergies (Santello et al., 1998) and soft robotics. By combining the concept of hand postural synergies and soft robotics, the result is an artificial system actuated with only one motor, which implements the first human hand synergy in grasping in free-hand motion. At the same time, the SH, is also adaptable and robust, and hence able to grasp different types of items. The prosthetic version, the SH-P, can be controlled using two EMG signals from a couple of agonistic–antagonistic (Ag–Aag) wrist or finger muscles.

The present study was designed to identify the myoelectric controller that could generate SH-P finger movements with the greatest reliability and degree of similarity of finger movements of the native hand. Our investigation consisted of three steps. First, we quantified the kinematic consistency of SH-P movements across a large number of movements to evaluate the repeatability of its performance. The objective of this evaluation was twofold: (1) to assess the reliability of SH-P response to motor commands and (2) to ensure that the evaluation of similarity between SH-P and native finger movements would not be biased by random inconsistencies in SH-P response to motor commands. Second, we evaluated the most suitable body sites on the forearm for recording EMG defined in terms of SNR. As described for the first objective, this evaluation was necessary to ensure that myoelectric controllers could be reliably compared by using the best possible EMG signals as inputs to the controllers. Furthermore, high SNR of EMG signals contain greater information about modulation of muscle activity responsible for finger movements, and could, therefore, be better exploited by the myoelectric controllers. The third evaluation, which is the core objective of the present work, compared three myoelectric control algorithms. The optimal controller was defined as the algorithm that generated SH-P finger kinematics that best resembled finger kinematics of the native hand.

Although there is an extensive literature on myoelectric prosthesis controllers [early work dates back to the 50s as (Battye et al., 1955), for a review, see, e.g., Castellini et al. (2014)], literature on the use of EMG signals for the control of synergistic movements is moving its first steps. Within this growing framework, it is important to note that the aim of this work is to provide an assessment of myoelectric controller performance and kinematic behavior of a specific device, the SH-P. For these reasons, algorithms were specifically tailored to be used with the SH-P, although other commercial prostheses are endowed with similar controllers. Results of this paper represent a stepping stone toward more in-depth investigation on the extent to which the hardware and software of the SH-P can overcome limitations of existing hand prostheses.

## 2 Materials and Methods

### 2.1 Participants

We performed three experiments. Experiment 1 quantified the repeatability, reliability, and consistency of the SH-P movements. Experiment 2 was designed to identify the agonist–antagonist muscle pair characterized by the highest SNR. Experiment 3 quantified the EMG-to-position mapping algorithm that provided the best correspondence between finger movements of the native hand and SH-P.

*Experiment 1* did not involve testing of human subjects as the SH-P was controlled by artificial commands (see below). For *Experiment 2*, we tested four able-bodied volunteers (1 female, 3 males; age range: 21–26 years, mean ± SD: 22.5 ± 2.06). For *Experiment 3*, we tested fourteen able-bodied volunteers (5 females, 9 males; age range: 18–27 years, mean ± SD: 20.36 ± 2.43).

All subjects were tested on their dominant hand (right hand; self-reported hand dominance). All participants were naive to the experimental purpose of the study and had no history of neuromuscular disorders. Before data collection, subjects signed an informed consent to participate in the experiment. The experimental protocols were approved by the Institutional Review Board at Arizona State University in accordance with the Declaration of Helsinki.

### 2.2 Apparatus

**Figure 1:**
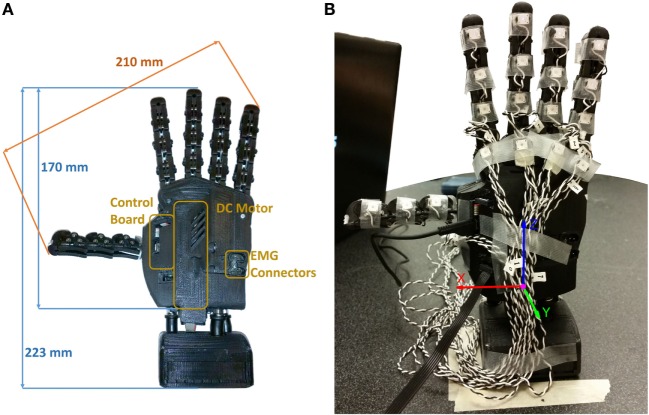
**SH-P used for Experiments 1 and 3**. **(A)** Dimensions of SH-P and the main components. **(B)** Markerization protocol (Experiment 1). Note that only the markers placed on the distal phalanges were used for the experiment, whereas additional markers in a triangular configuration were placed on the palm (not visible in this picture).

For the present investigation of myoelectric controller algorithms for hand prosthesis, we used the SoftHand-Pro (SH-P) (Figure 1). The SH-P is the prosthetic version of the Pisa/IIT SoftHand (SH), a robotic hand that had been designed for humanoid robots (Catalano et al., 2012, 2014).

The main design concept underlying the development of the SH (Catalano et al., 2012, 2014) is the construction of a robust, safe, low-cost, and simple robotic hand. The SH finger movements occur along the first hand postural synergy as defined by Santello et al. (1998), i.e., the first principal component extracted from static hand postures used to grasp a wide variety of imagined objects. This first postural synergy was implemented in the SH-P by combining it with a soft-robotics approach, through which a reference position of a “virtual hand” attracts the real hand, thus resulting in the concept of “soft synergy” (Bicchi et al., 2011). The soft-synergy approach enables a better control of the interaction forces between the hand and the grasped object, thus allowing the SH-P to grasp a large variety of items.

Another important feature of the SH design is that it is underactuated (Birglen et al., 2007). Specifically, the SH has 19 DOFs, 4 on each finger, and 3 on the thumb, but they are all actuated by only one motor (Figure 1). This feature effectively reduces weight, costs, and control complexity, thus making it an ideal candidate for prosthetics applications, since it only requires two EMG signals from an agonist–antagonist muscle pair (Zhao et al., 2015). The actuator is a DC motor that pulls a tendon running across the “phalanges” through a system of pulleys. Movements of the SH are controlled using a PID controller, which takes as inputs the desired angular position of the motor and the real position measured by a 12-bit magnetic encoder (resolution: 0.0875°) applied to the motor. All the structural components of the palm are built with rapid prototyping material (ABSplus – Stratasys), all the phalanges are fabricated using injection molding except for the “metacarpo-phalangeal” joint of the thumb, which is fabricated using a computer numeric control machine.

The original design of the SH was modified for prosthetic applications. Specifically, the overall size of the device was reduced to obtain the size of an average human male hand. The electronics were reduced in size and modified to enable interfacing with commercial EMG electrodes (Otto Bock, Germany). The electronics, previously placed at the base of the hand, was moved to the back to obtain a more compact design (Figure 1). The original SH motor (15-W Maxon motor RE-max-21 24 V) was substituted with a smaller and lower voltage motor (14-W Maxon motor DCX-22-S 12 V) and battery. The dimensions of SH-P used for the present work are 210 mm from the tip of the thumb to the tip of the little finger, and 170 mm from the base of the palm to the tip of the middle finger (225 mm relative to the wrist interface). The thickness of the palm is different between the thumb and the little finger sides of the hand, i.e., 40 mm on the little finger side, where the EMG connectors are placed, and 53 mm at the motor location (the thumb side), respectively. The SH-P is powered using an 11.1-V AR.Drone 2.0 HD Battery 1500 mAh (Parrot) connected via standard cable connection. For further information about the technical characteristics of the SH-P, the reader is referred to Catalano et al. (2014).

### 2.3 Experiments

#### 2.3.1 Experiment 1: Testing Repeatability of SH-P Finger Kinematics

Experiment 1 was designed to assess the extent to which the SH-P could be used for assistive or rehabilitation purposes by quantifying the repeatability, reliability, and consistency of the SH-P finger movements in free-hand motions. As myoelectric control through a human user could contribute to across-trial variability in SH-P finger kinematics, this experiment removed this potential confound by driving the SH-P through artificial commands. Thus, this design allowed us to isolate the causes of potential variability of SH-P kinematics to its mechanical design.

##### 2.3.1.1 Apparatus

The SH-P was placed vertically, fixed to the table through the wrist interface, and connected via a USB-A to micro USB-B cable to the laptop that was used to send commands to the SH-P and collect data from the on-board hand encoder. We used an active motion tracking system (PhaseSpace Motion Capture system, PhaseSpace Inc.) to record SH-P finger movements through 10 cameras (optical resolution: 3600 × 3600 – impressive sub-pixel resolution: 360,000 × 360,000 at 960 Hz). We placed a total of 22 light-weight infrared active LED markers (jitter <0.5 mm) on the SH-P, including each SH-P “phalanx” and “metacarpo-phalangeal” joints, and on the palm to create a local reference system (Figure 1). For the purpose of this experiment, we analyzed only the markers on the distal phalanges and palm. The orientation of the hand was defined to minimize marker occlusion and maximize capture of all markers by at least 3 cameras for at least 90% of the trial duration.

##### 2.3.1.2 Experimental Protocol

Custom software was used to control the timing of SH-P finger position (update rate: 8 Hz). We tested a total of sixty-three closing–opening movement cycles that were performed at increasing velocities. The SH-P velocities were expressed in motor steps per sample and ranged from 10 to 1000 steps/sample. We tested twenty-one SH-P velocities (3 trials per target velocity), corresponding to 10 up to 200 steps/sample with increments of 10 steps/sample, whereas the twenty-first velocity was 1000 steps/sample. Note that 10 steps/sample is the smallest step resolution that can produce an observable change in SH-P finger motion. Although the 1000 step/sample velocity does not capture a realistic SH-P finger velocity associated with activities of daily living, being it faster than the usual velocities, the SH-P moves during the normal usage; this velocity was chosen as a stress test of the SH-P hardware. We recorded three-dimensional position of the active markers on the SH-P during all trials.

##### 2.3.1.3 Data Processing and Analysis

When markers occlusion occurred, marker position data were interpolated. We found that most of the marker occlusions occurred at the end of the movement, i.e., at closed fist posture. Therefore, all makers, hence finger kinematics, could be reliably tracked from the initial position (fully open hand) and throughout the entire closing movement, with the exception of a small subset of markers when the hand was fully closed. The next step of kinematic data processing was to transform all marker positions defined with respect to an inertial system of reference, in the local reference frame, defined through the markers attached to the palm of the hand in a triangular arrangement. The new reference frame was defined in order to have the x-axis directed from the little finger side of the hand to the thumb one, with its origin being the center of the base of the triangle. The z-axis was directed from the origin to the upper vertex of the triangle, whereas the y-axis, according to right hand rule, pointed inside the hand (Figure 1). The path of the fingertips was visualized in three ways: (1) all paths defined by the markers on the fingers were plotted in 3-dimensional space; (2) the value of the three-dimensional position of each fingertip was defined with respect to the position of the motor of the SH-P, averaged across trials, and the mean ± SD values for each fingertip position were plotted; and (3) the position of each fingertip was plotted against time-normalized trial duration. Using the data processed as described in (2), for each fingertip, we computed the root mean square difference (RMSD) between fingertip position from individual trials and the mean of fingertip position computed across all trials as follows:

$$\text{RMSD}=\sqrt{\frac{\sum_{t=1}^{n}{\left({ŷ}_{t}-y_{t}\right)}^{2}}{n}}$$

where *ŷ_t_* is the sample of the single trial, *y_t_* is the mean of the sample averaged across all trials.

#### 2.3.2 Experiment 2: Analysis of EMG Signal-to-Noise Ratio

The goal of this experiment was to identify the muscle pair characterized by the highest signal-to-noise (SNR) ratio as a means to reliably compare the performance of three myoelectric controller algorithms (Experiment 3). To allow a realistic comparison with using myoelectric controllers in individuals with upper-limb loss, we did not perform an exhaustive search of target muscles to record EMG from to try to minimize cross talk among adjacent wrist and extrinsic finger muscles, e.g., specific finger compartments within the target finger muscle. Rather, we focused on selecting an agonist–antagonist muscle pair whose EMG activity (1) was characterized by the greatest SNR and (2) could be reliably elicited and recorded across trials when performing reach-to-grasp movements.

##### 2.3.2.1 Apparatus

We used two surface EMG electrodes that are commonly used for myoelectric prostheses (linear-proportional 13E200 MYOBOCK electrodes, Otto Bock, Germany). These electrodes are equipped with a logarithmic sensitivity adjustment and high common-mode rejection in the low frequency range (>100 dB at 50 Hz). The electrodes were applied in correspondence of the wrist and finger muscles. We targeted two wrist muscles (m. flexor carpi radialis, FCR, and m. extensor carpi ulnaris, ECU) responsible for wrist flexion and extension, respectively, and two extrinsic finger muscles (m. flexor digitorum superficialis, FDS, and m. EDC) responsible for finger flexion and extension, respectively. We used a LabView program (National Instruments) to record EMG data (sampling rate: 1 kHz).

##### 2.3.2.2 Experimental Protocol

We asked subjects to reach, grasp, lift, and replace a cylindrical glass bottle (weight: 400 g) whose position was aligned with the right shoulder of the subject on the sagittal plane. In the start position, the subject was instructed to open the hand with the palm parallel to the sagittal plane of the body. The distance between the subject’s hand start position and the bottle was 25 cm. We asked subjects to perform two block of reach-go-grasp trials. In the first block (15 trials), subjects performed slow reach-to-grasp movements to be completed within 13 s (reach onset to object grasp). In the second block (5 trials), subjects were asked to perform the same movement, but at a faster speed such that the whole movement had to be completed within 7 s. In each reach-to-grasp trial, subjects were asked to move the hand in a direction perpendicular to her/his body to reach the cylinder. The experimenter gave a “go” signal to cue subjects to start the reach onset. In our instructions to subjects, we emphasized that reach-to-grasp movement should be performed in a natural fashion, i.e., similar to the way one reaches for a cup on a desk. Once the hand had reached the bottle, subjects were asked to grasp, lift (~1 cm height), hold (~1 s), and replace it on the table and place the hand back to its stat position. We used the same EMG electrode gain for both muscle pairs.

##### 2.3.2.3 Data Processing and Analysis

The data were analyzed by computing the SNR of the EMG signals during the two blocks of reach-to-grasp action and isometric maximal voluntary contractions as

$${\text{SNR}}_{\text{dB}}=10{\text{ log}}_{10}\frac{\text{RMS}\left(\mathrm{Signal}\right)}{\text{RMS}\left(\mathrm{Noise}\right)}$$

where the noise level was measured at rest in between trials. Noise measurement and SNR computation were performed as described in Solnik et al. (2008). We used the Wilcoxon rank sum test to determine statistically significant differences in the SNR of the two muscle pairs. We computed a pairwise comparison between SNR from finger muscles EMG versus SNR from wrist muscles EMG by pooling all trials (slow and fast) and extensor and flexor muscles within each category.

#### 2.3.3 Experiment 3: Identification of Optimal Myocontroller Algorithm

The goal of this experiment was to find the most effective EMG mapping algorithm that could generate SH-P finger kinematics during reach-to-grasp movements that best resembled native hand kinematics. To attain this objective, we compared the performance of three EMG-to-SH-P movement mapping algorithms, in terms of effectiveness of movement activation (i.e., the ability of a given algorithm to generate SH-P finger motion) and similarity in the kinematics of SH-P and native hand.

##### 2.3.3.1 Apparatus

**Figure 2:**
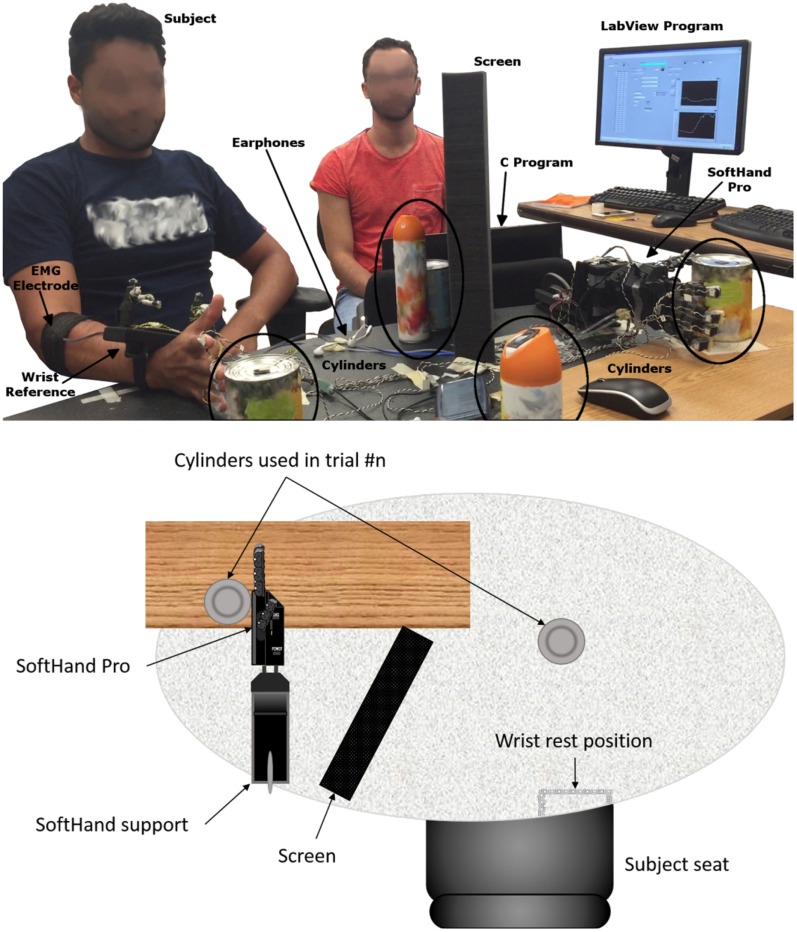
***Experiment 3*: experimental setup**.

We used three pairs of identical cylindrical objects (cans) with different radii and weight (52 mm and 250 g; 74 mm and 450 g; 85 mm and 600 g). One of the objects in each pair was to be grasped by the subject with his/her native hand, whereas the object was grasped by the SH-P (Figure 2). The SH-P grasping movement was controlled by two EMG electrodes placed on the subjects forearm as he/she reached to grasp the target object. The object to be grasped by the subject was positioned on the table surface and aligned with the sagittal plane passing through the subject’s right shoulder to avoid large wrist movements. The reach distance between start hand position and target object was 25 cm. The object to be grasped by the SH-P was placed within the palm of the SH-P. View of the SH-P during the reach-to-grasp movement with the native hand was blocked by a screen. To further prevent subjects from getting distracted, they wore headphones with white noise. We used the same motion tracking system used in Experiment 1 to record SH-P and native hand kinematics. The SH-P and native hand were outfitted with markers. For the SH-P, we placed markers on each fingertip (on the back of the third phalanx), and three reference markers on the wrist as done in Experiment 1. On the subject’s hand, we placed a marker on the tip of each nail and eight markers on a 3D printed wrist structure taped on the subjects wrist to define a local reference frame [for details, see Gabiccini et al. (2013)]. Markers were also placed on each of the target objects. As done in Experiment 1, the hand and the objects were positioned to ensure that markers could be viewed by at least 3 cameras. The SH-P parameters were managed through custom software by a laptop. Custom software was also used to record SH-P motor position data. A LabView program (National Instruments), running on a second computer, was used to record EMG and motion tracking data. Data collection by the two computers was synchronized offline via software at 90 Hz.

##### 2.3.3.2 Experimental Protocol

**Figure 3:**
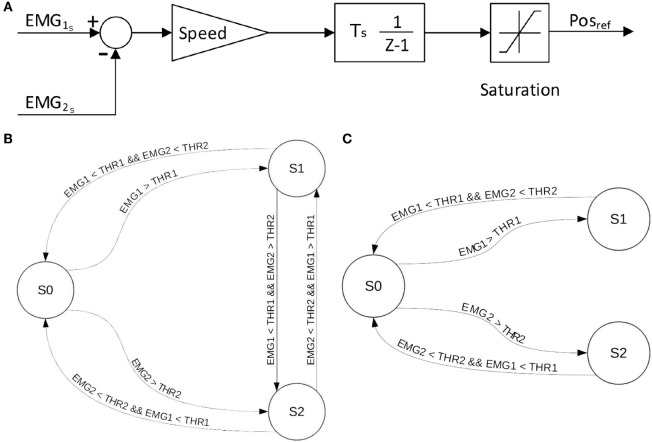
**EMG-to-position algorithms implemented on the SH-P**. EMG-differential **(A)**, EMG-FCFS **(B)**, and EMG-FCFS-Advanced **(C)**. EMG1 and EMG2 denote the two signals acquired from the agonist–antagonist muscle pair. THR1 and THR2 denote the threshold for EMG1 and EMG2, respectively. S0, S1, and S2 denote the steady position, closing movement, and opening movement, respectively.

Before performing the reach-to-grasp experiment and test each of three myoelectric algorithms (see below), the SH-P was calibrated by asking subjects to perform a maximal voluntary isometric contraction during finger flexion and extension while recording EMG of each muscle from the target muscle pair (FDS and EDC, respectively). These muscles had been identified in Experiment 2 as those with the highest SNR. The EMG amplitude recorded during calibration is used by the controller to define the threshold EMG amplitude above which finger motion occurs as well as the upper limit of EMG amplitude. The SH-P was mounted horizontally on a support fixed to the table (with the palm parallel to a vertical plane, thumb up) (Figure 2). To increase the sensitivity of the SH-P to EMG signals, the EMG threshold amplitude obtained with the calibration phase obtained through maximal voluntary contractions was reduced to 8 and 14% of the maximum FDS and EDC EMG amplitude recorded. We used the same EMG electrodes and recording procedures we used in Experiment 2. During calibration, the subject sat in the same position used during the experiment. Once the hand was calibrated, the subject started the experiment. Subjects performed one block of 60 trials each for each of the three myoelectric controller algorithms with a 5-min break between blocks. We tested three EMG controller algorithms. On each trial, custom software was used to select in a pseudo-random order, a given myoelectric controller algorithm, before the start of data collection start. Each algorithm was presented the same number of times. We tested the following algorithms:

1. *EMG differential*: this EMG-to-motor position mapping uses the difference between the EMG signals recorded by the two EMG electrodes minus the value of their respective thresholds. The sign of the EMG amplitude difference dictates the SH-P finger movement direction, whereas the amplitude of the difference defines finger movement velocity (Figure 3A).
2. *EMG-first come first served (FCFS)*: the first EMG signal that goes over 10% of the maximum value determined by the calibration procedures defines the direction of SH-P finger movement whereas its amplitude defines movement velocity. The direction of the movement is inverted if the amplitude of the leading EMG signal goes below a threshold while the amplitude of the other EMG signal is above the threshold (Figure 3B).
3. *EMG-FCFS-Advanced*: this algorithm avoids involuntary inversion of the movement direction by allowing it only if the amplitude of both EMG signals goes below the threshold (Figure 3C).

**Figure 4:**
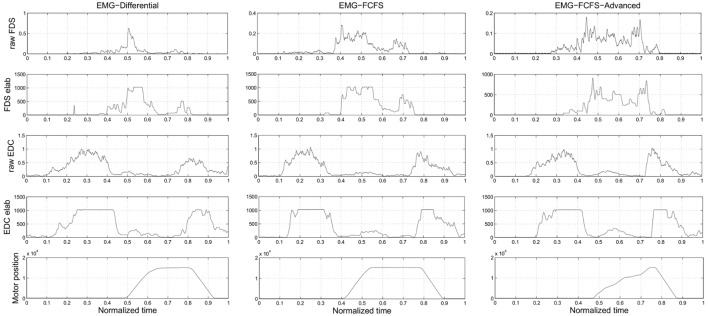
***Experiment 3*: this picture presents how the different algorithms manage the EMG signals**. The plots of the real EMG signals, the EMG signals after the elaboration, and the application of the thresholds inside the SH-P firmware and the motor positions are presented. The presented data are obtained during a real trial of Experiment 3.

In Figure 4, it is possible to observe how the different algorithms manage the recorded EMG signals on the SH-P.

Each of the three algorithms were presented in two ways that differed depending on whether a modulation of the proportional gain, driven by the ratio between the two EMG signals, was implemented. Thus, for each subject, we considered trials with versus without the EMG-driven P-gain modulation (see above; Table 1). For the P-gain modulation trials, the proportional gain of the PID controller of the position control of the motor increased with increasing amplitude of the EMG signal with the smallest amplitude, normalized to the EMG signal highest amplitude. Upper and lower bounds for the proportional gain were estimated through pilot data by selecting the range of values in which the SH-P moved smoothly until reaching the reference position. SH-P movement velocity and impedance were modified by changing the proportional gain value. The reach-to-grasp task instructions and experimental procedures were the same as those described for Experiment 2; however, no constraint was imposed on the time required to complete the task. We instructed the subjects to contact the object and exert grip with all the fingers. More specifically, participants were required to keep their hand with the palm parallel to the sagittal plane and with the line from the wrist to the middle finger parallel to the transverse plane. During the experiment, subjects’ movements were visually inspected by experimenter in order to verify that the aforementioned instructions were correctly executed. If the experimenter noted any major deviation from the desired behavior, the trial was repeated. As subjects reached toward the object, the SH-P moved accordingly based on the EMG-to-position mapping selected for that trial. The three object sizes were presented in a pseudo-random order and for an equal number of trials for each algorithm.

**Table 1:** ***Experiment 3*: activation rate with the different algorithms, considering (W PGM) and discarding (W/o PGM) the proportional gain modulation**.

Subj.	EMG-diff			EMG-FCFS			EMG-FCFS-adv		
	Tot. (%)	W/o PGM (%)	W PGM (%)	Tot. (%)	W/o PGM (%)	W PGM (%)	Tot. (%)	W/o PGM (%)	W PGM (%)
1	98.33	96.67	100.00	15.00	10.00	20.00	40.00	26.67	53.33
2	68.33	66.67	70.00	0.00	0.00	0.00	1.67	3.33	0.00
3	88.33	86.67	90.00	41.67	46.67	36.67	30.00	33.33	26.67
4	5.00	6.67	3.33	0.00	0.00	0.00	11.67	23.33	0.00
5	86.67	80.00	93.33	0.00	0.00	0.00	3.33	0.00	6.67
6	26.67	36.67	16.67	1.67	3.33	0.00	3.33	6.67	0.00
7	100.00	100.00	100.00	10.00	10.00	10.00	5.00	0.00	10.00
8	8.33	16.67	0.00	5.00	10.00	0.00	21.67	26.67	16.67
9	0.00	0.00	0.00	5.00	10.00	0.00	5.00	0.00	10.00
10	0.00	0.00	0.00	3.33	0.00	6.67	3.33	3.33	3.33
11	88.33	83.33	93.33	40.00	43.33	36.67	55.00	50.00	60.00
12	91.67	90.00	93.33	3.33	3.33	3.33	15.00	13.33	16.67
13	58.33	63.33	53.33	3.33	3.33	3.33	15.00	13.33	16.67
14	100.00	100.00	100.00	13.33	16.67	10.00	0.00	0.00	0.00
Mean	58.57	59.05	58.09	10.12	11.19	9.05	15.00	14.29	15.72

*Motion was considered successfully activated when SH-P motor position reached or overcame 25% of the whole range*.

##### 2.3.3.3 Data Processing and Analysis

For each algorithm, our analysis focused on estimating (1) how many times the subject was able to activate the SH-P movements with a normal reach-to-grasp action and (2) quantifying the similarity between the SH-P and native hand finger kinematics. To define the extent to which SH-P responded to EMG-based commands, we computed the number of times that EMG signals could trigger SH-P finger movements. To verify whether EMG signals triggered SH-P finger movement, we examined the angular position of the motor. Movement activation was defined as the angular position above the minimum input value required to reach the 25% of the total SH-P movement range. The threshold in the position of the motor was the only parameter used to discriminate trials with successful from failed activation of the SH-P. Note that the analysis of the activation rate does not take in account eventual delays or deactivations after the threshold crossing; this last event will impact the index shown above. For the identification of the optimal myoelectric controller algorithm, we used trials in which SH-P finger movements could be generated by EMG signals and quantified the similarity of the paths of the SH-P and native hand fingertips during reach-to-grasp movements. This analysis consisted of computing a numeric index between 0 and 1 corresponding to no similarity and identical kinematics between finger movements in the SH-P and native hand, respectively. The vector distances (three components) between the thumb and each of the four fingers were computed for both the SH-P and the native hand. These values were computed for each sample of each trial and normalized by dividing each component of the vector by the amplitude of the vector itself. This procedure removes the effect of different sizes of the SH-P and native hand on the comparison of their vector distances. We then computed the dot product between vectors pairs obtained from the native and artificial hand. The obtained four values, one for each thumb-finger vector, were summed and divided by 4 to compute the average similarity index. The mean similarity index was computed for each myoelectric controller algorithm.

## 3 Results

### 3.1 Experiment 1

**Figure 5:**
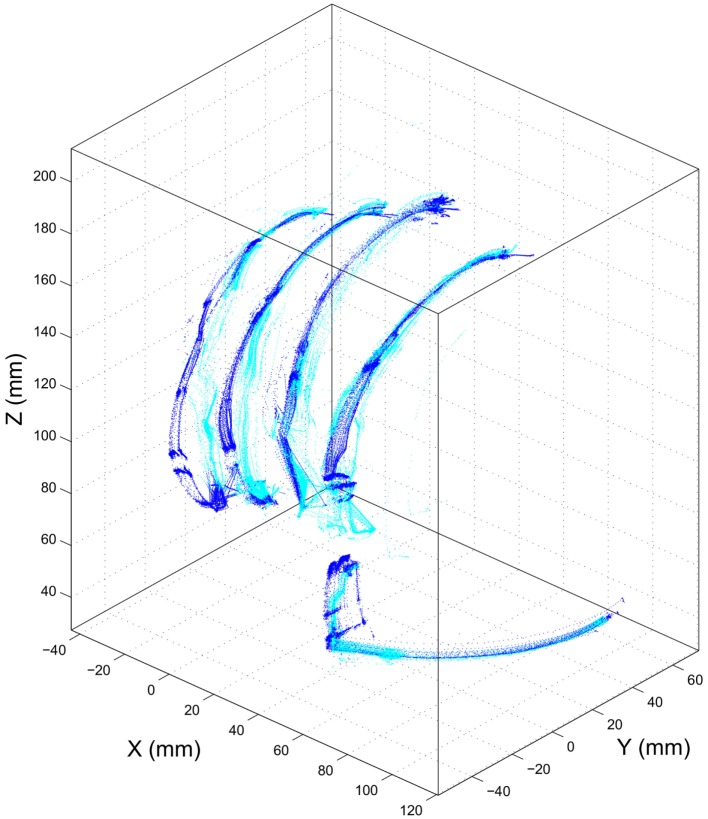
***Experiment 1*: path of SH-P fingertip in three-dimensional coordinates**. Dark and light blue traces denote hand closing and opening paths, respectively.

**Figure 6:**
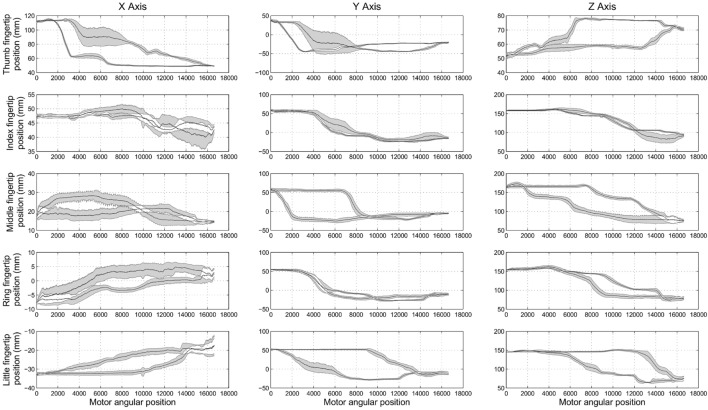
***Experiment 1*: spatial position of SH-P fingertips (expressed in the spatial coordinates x, y, and z) w.r.t. the angular position of the motor**. Black traces in the subplots are the mean closing and opening paths averaged across trials, and gray areas denote the SD of the mean path.

Experiment 1 was designed to assess the consistency of SH-P finger movements across trials and velocities. Figure 5 shows the three-dimensional coordinates of each fingertip of the SH-P recorded during multiple hand opening–closing cycles. It can be seen that the fingertip paths during hand opening and closing (dark and light data points) are fairly consistent across movement cycles. To further visualize the consistency of fingertip paths, Figure 6 shows these data projected on the three axes of the reference frame and expressed with respect to the motor angular position.

**Figure 7:**
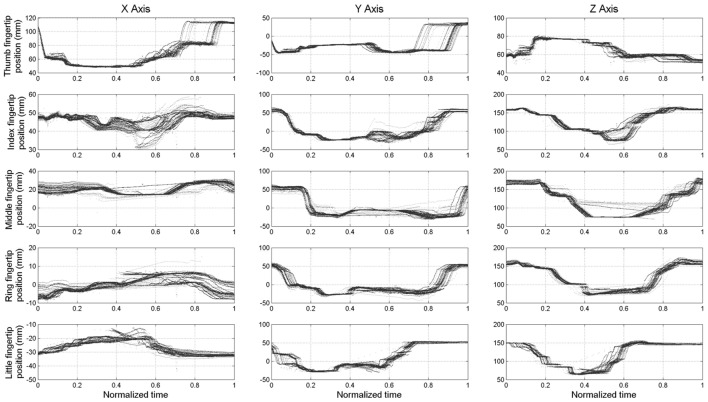
***Experiment 1*: spatial position of the fingertips (expressed in spatial coordinates x, y, and z) w.r.t. the time normalized on trial duration**.

The maximum root mean square difference (RMSD) across all paths shown in Figure 6 were 9.4 mm for the thumb (x-axis), 5.7 mm for the index finger (x-axis), 6.5 mm for the middle finger (z-axis), 4.4 mm for the ring finger (y-axis), and 5 mm for the little finger (y-axis). A high degree of consistency in fingertip paths can also be appreciated across different movement velocities when plotting finger paths as a function of normalized time (Figure 7).

Some shifts with respect to time and motor position, considered as offsets, can be observed. This is more evident in the final phase of the opening of the thumb (x-axis and y-axis) and in the central phase of the middle finger (z-axis). The data plotted in Figure 6 also show hysteresis in the finger path during hand opening versus closing movements. However, this hysteresis, which is due to the elastic elements embedded in the joints that enable the passive opening of the SH-P, does not affect finger path consistency across trials and movement velocities. The small RMSE of fingertip paths indicate that SH-P finger movements are very consistent across multiple hand opening–closing cycles and movement velocities.

### 3.2 Experiment 2

**Figure 8:**
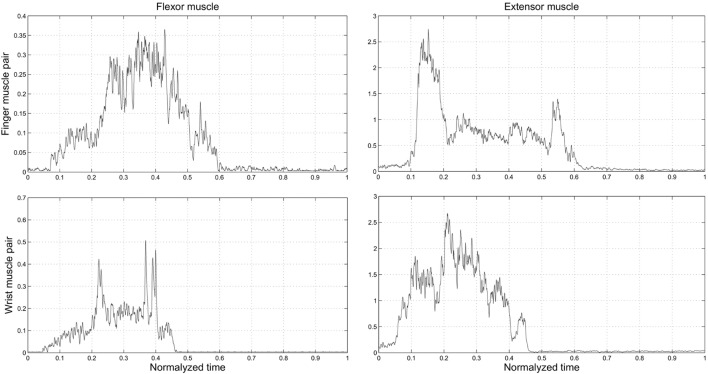
***Experiment 2*: time course of EMG activity from finger and wrist muscles (top and bottom row, respectively) as a function of normalized time on the trial duration**. “0” and “1” denote the starting moment of the movement of reach and the end of the trial within the predefined time, when the hand was back in starting position, respectively.

The objective of Experiment 2 was to determine the muscle pair characterized by the highest signal-to-noise ratio (SNR) to be used for comparing myoelectric controllers in Experiment 3. We found that SNR was significantly greater for the finger than wrist muscles EMG signals (27.73 and 21.88 dB; Wilcoxon rank sum test: P = 0.0015). Therefore, we chose to use the FDS-EDC muscle pair for Experiment 3. This choice was also motivated by the objective of allowing for a more natural and intuitive myoelectric control of the SH-P. Indeed, during a reach-to-grasp action, the time course of finger muscles’ EMG signals is closely related to the time course of the SH-P finger motion (Figure 8). Specifically, the two EDC EMG peaks correspond with SH-P opening during pre-grasp and at object release at the end of the trial. Similarly, the FDS EMG signal exhibits a reciprocal activation pattern relative to EDC, i.e., most of its EMG activity is found between the EDC peaks during SH-P closing. In contrast, this correspondence between EMG patterns and SH-P finger motion was not being captured by wrist muscles EMG.

### 3.3 Experiment 3

Experiment 3 was designed to identify the myoelectric controller algorithm that could elicit SH-P movements with the greatest degree of consistency and whose kinematics best resembled finger kinematics of the native hand.

Table 1 shows the rate at which each myoelectric controller algorithm could elicit SH-P finger movements. The EMG mapping algorithm characterized by the highest activation rate was the EMG-differential (58.57%), whereas the EMG-FCFS algorithm was characterized by the lowest activation rate (10.12%).

However, even the best performing algorithm failed to generate SH-P movement in a consistent fashion. Further examination of EMG data revealed that such failure occurred more often in subjects who exhibited a lower ability to selectively activate one of the two muscles independently from the other and/or to inhibit muscle activity when switching from hand opening to closing. An important observation was that often the SH-P responded better to hand-closing than hand-opening EMG signals. This, however, happened significantly less often when EMG signals were mapped to SH-P movements through the EMG-differential algorithm. This could have been due to the fact that, during the object release, subjects not always fully extended the fingers but rather relaxed them just enough to trigger object release, but not a full extension of the SH-P fingers. Thus, the EMG amplitude associated with this action might not have been sufficient to trigger opening of the SH-P.

Note that SH-P activation rates were not affected by the implementation of the EMG-driven proportional gain modulation (PGM, Table 1; chi-squared test = P > 0.05).

Table 2 shows the similarity index computed on the SH-P and native hand kinematics using only the trials that successfully elicited SH-P movements as described above.

**Table 2:** ***Experiment 3*: kinematic similarity index between SH-P and real user movements, for the different mapping algorithms, considering (W PGM) and discarding (W/o PGM) the proportional gain modulation**.

Subj.	EMG-diff		EMG-FCFS		EMG-FCFS-adv	
	W/o PGM	W PGM	W/o PGM	W PGM	W/o PGM	W PGM
1	0.8840	0.8848	0.9337	0.9381	0.9598	0.8242
2	0.8157	0.8446	–	–	0.9014	–
3	0.8306	0.8369	0.8144	0.8398	0.7844	0.8094
4	0.7565	0.7029	–	–	0.8646	–
5	0.8218	0.8489	–	–	–	0.7557
6	0.7888	0.8348	0.8086	–	0.9357	–
7	0.7344	0.7489	0.6389	0.7271	–	0.6768
8	0.7985	–	0.8441	–	0.8394	0.8318
9	–	–	0.9284	–	–	0.7624
10	–	–	–	0.8296	0.9050	0.7624
11	0.8225	0.8027	0.9075	0.8256	0.8593	0.8716
12	0.8833	0.8868	0.9784	0.8743	0.8942	0.9091
13	0.6976	0.6907	0.6506	0.8224	0.8530	0.7845
14	0.8885	0.8914	0.9121	0.9173	–	–
Mean	0.8102	0.8158	0.8417	0.8468	0.8797	0.7988

*A score close to 1 indicates a high level of similarity. The absent data are relative to the conditions with activation rate 0%. Indeed, in this analysis, we only considered trials where the SH-P was activated*.

All EMG mapping algorithms resulted in SH-P kinematics that was very similar to native hand kinematics (range of similarity index: 0.80–0.88). We found no statistically significant difference in the similarity index across the three EMG myoelectric controller algorithms. Taking into account the significantly greater activation rate for the EMG-differential algorithm, we conclude that this EMG mapping algorithm is preferable since it ensures both a more reliable activation of SH-P movement in response to EMG signals while resembling native hand kinematics.

## 4 Discussion

The overarching goal of our three experiments was to characterize the performance of a myoelectric hand prosthesis, the SoftHand-Pro, to assess its feasibility as an assistive and rehabilitative tool. Specifically, we sought to quantify the extent to which the SH-P finger movements resembled human finger movements. We reasoned that this factor should be evaluated as it might contribute to the extent to which individuals with upper-limb loss might accept the SH-P as a prosthetic device. To achieve this objective, we also quantified the consistency of SH-P finger movements and identified the muscles that should be used to extract EMG signals to control the SH-P. Below, we discuss our results and future research directions.

### 4.1 The SH-P Mechanical Design Enables Repeatable Finger Movement Kinematics

Successful control and performance of myoelectric prostheses rely on three factors: (1) the intrinsic properties of the terminal device (i.e., hardware), which affect how it responds to given EMG signals, (2) how EMG signals are processed (i.e., software) to generate motion of the terminal device, and (3) the extent to which the user and his/her motor commands (extracted through EMG signals) can adapt to the terminal devices hardware and software characteristics. When studying a human subject “in the loop,” these three factors interact in complex ways, thus making it difficult to understand the role of each factor on the terminal device performance. To ensure that we could identify the role of each of these three factors independently, we removed the potential effect of (3) from (1) by driving multiple SH-P movement cycles through artificial, rather than EMG, control signals (Experiment 1). We should note that, although previous studies have examined the performance of the SH and SH-P through myoelectric control (Godfrey et al., 2013, 2014; Ajoudani et al., 2014; Bonilla et al., 2014; Zhao et al., 2015), the present study is the first to examine repeatability of finger kinematics without the confound of trial-to-trial and/or between-subject variability of myoelectrical signals.

We found that SH-P finger movements were highly consistent across movement cycles and velocities (Figures 5–7). Thus, the soft synergy-based design allows the control of 19 degrees of freedom through the action of one motor without compromising the reliability of multi-finger motion. This is an important result when considering the needs of individuals with upper-limb loss to perform repeatable hand movements through EMG control. In particular, relying on repeatable kinematics for given EMG inputs should facilitate the adaptation of motor commands/EMG and learning to use the SH-P effectively.

### 4.2 Finger Muscles EMG as Control Signals for the SH-P

To control multi-fingered hand prostheses through EMG signals, the classic approach is to employ signals collected from multiple muscles and then to use these signals as inputs to ML systems. This approach results in creating a direct mapping from multi-EMG patterns and multi-DOF devices (Castellini et al., 2009; Tenore et al., 2009; Ison and Artemiadis, 2014). In contrast, the design of the SH-P enables the use of a minimal number of EMG signals (two) to control one degree of actuation. However, thanks to its adaptability, the SH-P can exploit the external environment as a multiplier of its DOF, thus enabling the performance of a wide of range of activities of daily living (Godfrey et al., 2013; Ajoudani et al., 2014; Centro di Ricerca Enrico Piaggio et al., 2016; Santello et al., 2016).

Before, we could address the question of what myoelectric controller optimizes SH-P performance, we had to identify the muscle pair whose EMG signals was characterized by the greatest signal-to-noise ratio (SNR). As noted for the testing of SH-P finger kinematics repeatability, these questions were not addressed in previous SH-P studies. We found that SNR was significantly greater in finger than wrist muscles. Although the goal of our study was not to perform a systematic evaluation of SNR across many pairs of finger or wrist muscle pairs, this result was important to rule out a potential confound in our evaluation of myoelectric controllers, i.e., negatively biasing the responsiveness of the SH-P to EMG signals or performance of a given myoelectric controller due to using EMG signals with poor SNR. Our EMG data also suggest that, because of the close correspondence between finger flexor/extensor EMG activity patterns and hand opening–closing (Figure 8), residual finger muscles – if available, and if the quality of EMG signal is acceptable – should be targeted for myoelectric control of hand prostheses. This recommendation is consistent with the above goal of enabling individuals with upper-limb loss control, a terminal device, in a way that closely mimics how they would have controlled their own hand, which may contribute to their acceptance of the hand prosthesis.

### 4.3 Defining the Myoelectric Controller for Optimal Reliability and SH-P Performance

Experiment 3 compared the effect of three EMG mapping algorithms for controlling SH-P finger motion during reach-to-grasp. This evaluation was based on the extraction of EMG signals from subjects using their native hand (as done for Experiment 2), while the SH-P was fixed in the proximity to a sensorized object. We found that the EMG-differential (Figure 3) was the most reliable algorithm for activating SH-P finger motion (59% of trials; Table 1). However, analysis of similarity of SH-P and native hand kinematics revealed that the EMG-FCFS-Advanced algorithm elicited the best performance (Table 2). As no significant statistical difference was found across the three myoelectric controller algorithms, and taking into account the greater ability of activating SH-P finger motion, we conclude that the EMG-differential algorithm should be used for future studies of SH-P prosthetic applications.

The superiority of the EMG-differential algorithm over the other two algorithms can be explained taking into account the criteria underlying each EMG-to-position algorithm. The EMG-FCFS and the EMG-FCFS-Advanced algorithms both rely on the existence of an EMG threshold. Specifically, the threshold is used to manage the signals by choosing the leading one and the direction of the movement. However, this threshold cannot be adjusted while operating the SH-P to guarantee the correct functioning of the algorithm. For this work, we selected the thresholds after analyzing previous recorded EMG signals path (during Experiment 2) so as to permit to the SH-P to move also in presence of weak EMG signals. The choice of a lower threshold would have resulted negative effects due to the noise recorded by the sEMG electrodes. This feature can account for the very low percentage of SH-P activations, since the signals generated during normal hand use did not result always strong enough to exceed the threshold. On the contrary, the EMG-differential control scheme does not use an EMG threshold. Therefore, any signal, even if it is small, could activate SH-P finger movements.

With regard to the EMG-differential, although this algorithm was characterized by the highest SH-P activation rate, it was not close to 100%. We believe that this is due to the fact that EMG signals were recorded during a natural reach-to-grasp movement with the native hand, rather than subjects attempting to move the SH-P through EMG signals. Furthermore, subjects were prevented from viewing the SH-P during the task and were, therefore, unaware of whether or how the SH-P moved. This was done to further remove the potential confound of EMG signals adapting to visual feedback of SH-P performance. Whereas this is a desirable phenomenon in terminal device training, for the purpose of the study, we had to remove this potential confound to focus on the ability of natural EMG activation patterns to elicit SH-P movements. Therefore, we believe that the percentage of trials characterized by SH-P activation could significantly increase by having subjects adapt their EMG activation patterns to viewing the SH-P during reach-to-grasp tasks.

Lastly, we also examined the effect of P-gain modulation on myoelectric controller performance as it can influence movement velocity, i.e., increasing the proportional gain of the PID controller of the SH-P could allow for a better trajectory tracking. We found that EMG drive P-gain modulation had no effect on the similarity between SH-P and native hand kinematics. Therefore, this technique could be used to enable other SH-P features, such as impedance control of the hand during interaction with objects (Ajoudani et al., 2014), without affecting the similarity with human finger kinematics.

## 5 Conclusion

Our results support the feasibility and potential of the SH-P as an effective hand prosthesis for individuals with upper-limb loss. Specifically, we found that the mechanical design of the SoftHand-Pro, combined with using EMG from finger muscles through the appropriate EMG myocontroller algorithm (EMG differential), enables a reliable motion of all SH-P digits through EMG recorded from only two finger muscles. A particularly encouraging result, which we believe is unique in the literature of hand prostheses, is that EMG-driven motion of SH-P fingers is very similar to native hand kinematics. This is important because, given the high abandonment rate of upper-limb prostheses, the movement and design anthropomorphism of the SH-P may contribute to greater acceptance of prostheses by individuals with upper-limb loss. We should also point out that the SH-P anthropomorphism offers great potential for using the SH-P as a novel assistive and sensorimotor rehabilitation device for individuals affected by neurological disorders, e.g., using the SH-P as a supernumerary limb (Prattichizzo et al., 2014).

While this work focuses on the control of SH-P, future work will address the usage of multi-EMG inputs [see Santello et al. (2016), for a review on this topic] for the control of multiple degrees of actuation, which were already implemented in a new purely robotic version of the SH (Della Santina et al., 2015).

## Author Contributions

SF, MB, AB, and MS designed the study. GG implemented the controllers of the SoftHand Pro. SF, MB, SJ, JN, and SB performed the experiments and data analysis. All authors contributed to writing the manuscript.

## Conflict of Interest Statement

The authors declare that the research was conducted in the absence of any commercial or financial relationships that could be construed as a potential conflict of interest.

## Abbreviations

Ag–Aag, agonist–antagonist; DOF, degree of freedom; ECU, extensor carpi ulnaris; EDC, extensor digitorum communis; EMG, electromyography; FCR, flexor carpi radialis; FDS, flexor digitorum superficialis; ML, machine learning; PGM, P-gain modulation; RMSD, root mean square difference; sEMG, surface electromyography; SH, Pisa/IIT SoftHand; SH-P, SoftHand Pro; SNR, signal-to-noise ratio.
